# Asymmetrical intraguild interactions with coyotes, red foxes, and domestic dogs may contribute to competitive exclusion of declining gray foxes

**DOI:** 10.1002/ece3.9074

**Published:** 2022-07-04

**Authors:** Dana J. Morin, Damon B. Lesmeister, Clayton K. Nielsen, Eric M. Schauber

**Affiliations:** ^1^ Department of Wildlife, Fisheries and Aquaculture Mississippi State University Mississippi State Mississippi USA; ^2^ Pacific Northwest Research Station, U.S. Forest Service and Department of Fisheries and Wildlife Oregon State University Corvallis Oregon USA; ^3^ Cooperative Wildlife Research Laboratory and Forestry Program Southern Illinois University Carbondale Illinois USA; ^4^ Illinois Natural History Survey, Prairie Research Institute University of Illinois Urbana‐Champaign Champaign Illinois USA

**Keywords:** biotic homogenization, co‐occurrence, interspecific competition, niche partitioning, trophic downgrading

## Abstract

Species coexistence is governed by availability of resources and intraguild interactions including strategies to reduce ecological overlap. Gray foxes are dietary generalist mesopredators expected to benefit from anthropogenic disturbance, but populations have declined across the midwestern USA, including severe local extirpation rates coinciding with high coyote and domestic dog occurrence and low red fox occurrence. We used data from a large‐scale camera trap survey in southern Illinois, USA to quantify intraguild spatial and temporal interactions among the canid guild including domestic dogs. We used a two‐species co‐occurrence model to make pairwise assessments of conditional occupancy and detection rates. We also estimated temporal activity overlap among species and fit a fixed‐effects hierarchical community occupancy model with the four canid species. We partitioned the posterior distributions to compare gray fox occupancy probabilities conditional on estimated state of combinations of other species to assess support for hypothesized interactions. We found no evidence of broadscale avoidance among native canids and conclude that spatial and temporal segregation were limited by ubiquitous human disturbance. Mean guild richness was two canid species at a site and gray fox occupancy was greater when any combination of sympatric canids was also present, setting the stage for competitive exclusion over time. Domestic dogs may amplify competitive interactions by increasing canid guild size to the detriment of gray foxes. Our results suggest that while human activities can benefit some mesopredators, other species such as gray foxes may serve as bellwethers for habitat degradation with trophic downgrading and continued anthropogenic homogenization.

## Graphical abstract

We directly assesses the effects of intraguild interactions and possible competitive exclusion among native canids and domestic dogs in a human‐dominated but rural landscape. The results demonstrate that human influences severely limit niche segregation among native canids potentially contributing to the decline of gray foxes, contrary to common assumptions that mesopredators benefit from human‐dominated landscapes, and highlighting the need to consider co‐occurrence and guild interactions over time as we assess the outcomes of trophic downgrading.

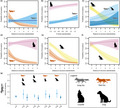

## 1 INTRODUCTION

Coexistence among sympatric competitors is facilitated by ecological niche separation (Gause, 1934; Schoener, 1974), including spatial, temporal, and dietary niche partitioning (Brown, 1989). However, all strategies reduce access to resources and interspecific competition imposes fitness costs that can reduce abundance (Creel & Creel, 1996) and lead to local extirpation (Hamel et al., 2013; Yackulic et al., 2014). The dynamics of competitive interactions over time can slowly result in range expansions for some species and contractions for others, even as these species appear to co‐occur over relatively short temporal scales. Furthermore, these processes are commonly amplified by anthropogenic influences (Farris et al., 2016; Schuette et al., 2013).

Gray foxes (*Urocyon cineoargenteus*) are found in a variety of habitat types and currently considered stable across North America (Roemer et al., 2016), but recent evidence indicates gray fox population declines in parts of their range (Allen et al., 2021; Bauder et al., 2020). Minimum harvest levels in the Midwestern United States decreased 10‐fold from the 1980s to 2015 (Association of Fish and Wildlife Agencies, 2019), including no individuals harvested in Illinois in 2015 (Bauder et al., 2021). Adjusted harvest‐based and hunter observation indices in Illinois provide concurrence with sharp population declines (Bauder et al., 2020, 2021) and the US Fish and Wildlife Service has been petitioned to list the prairie subspecies (*U. c. ocythous*) under the US Endangered Species Act (US Fish and Wildlife Service, 2012). Declines in gray fox populations in the Midwest coincided with increased in coyote (*Canis latrans*) populations and putatively decreased or stable red fox (*Vulpes vulpes*) populations (Bauder et al., 2020) while land cover has remained consistent (Allen et al., 2021; Walk et al., 2010), suggesting competitive interactions may be a contributing factor.

Strength of competition increases with ecological overlap among species including resource requirements, morphology, and phylogenetic relatedness (Brown & Wilson, 1956; Darwin, 1859; Dayan & Simberloff, 2005; MacArthur & Levins, 1964; Schoener, 1983). Intraguild interactions among canids are characterized by exploitative competition between species of similar size and diet, coupled with aggressive interference competition between species with moderate disparity in body size (Donadio & Buskirk, 2006; Palomares & Caro, 1999). Resource distribution and availability govern advantage in exploitative competition, and generalist species are better able to exploit heterogeneous environments, including human‐modified habitats (Clavel et al., 2011; MacArthur & Levins, 1967; Rodriguez et al., 2021). Resource availability also alters interference competition interactions, with scarcity intensifying aggression (Greenville et al., 2014). Furthermore, dispersion of resources can cause larger species to increase home range size (McNab, 1963), limiting refugia for smaller competitors.

In North America, gray and red foxes are generally similar in size and diet resulting in resource competition and spatial segregation when they co‐occur (Hockman & Chapman, 1983). Red foxes are common in areas with access to open habitat and woody cover and have adapted to anthropogenic habitats (Larivière & Pasitschniak‐Arts, 1996). As a result, red foxes have the widest geographical range of any carnivore (Hoffmann & Sillero‐Zubiri, 2016) and can exert intense exploitative competitive pressure on sympatric fox species utilizing similar food resources across a wide range of habitats (Hamel et al., 2013; Ilani, 1988). Gray foxes are forest specialist with shorter limbs adapted for climbing compared with more cursorial canids adapted for running speed (Feeney, 2000). Gray fox occupancy is expected to be greater in areas where forest cover is higher (Parsons et al., 2022) and agricultural interspersion is low (Lesmeister et al., 2015) compared with open grasslands and agricultural areas where red foxes are expected to be more abundant (Gosselink et al., 2007). A range‐wide analysis described a consistent negative association with red foxes demonstrating the influence of exploitative competition on gray fox occurrence (Allen et al., 2022).

Coyotes are larger than foxes and exert exploitative competition through dietary overlap (Cypher, 1993) and interference competition through fear‐mediated resource restriction and intraguild killing (Farias et al., 2005; Fedriani et al., 2000). No negative association was found between coyotes and gray foxes at a range‐wide scale (Allen et al., 2022), but spatial and temporal avoidance of coyotes was evident at smaller scales in urban areas (Fedriani et al., 2000; Parsons et al., 2022). Gray foxes often selected for brushy, or early successional habitats in rural areas (Cooper et al., 2012; Fritzell & Haroldson, 1982) increasing potential interactions with coyotes, but interactions may be mitigated by temporal avoidance or adequate hardwood tree cover providing refugia (Lesmeister et al., 2015; Parsons et al., 2022). Red foxes were found more frequently in grasslands than forests and appeared to avoid interference competition by selecting human‐associated habitats compared with the cover‐rich habitats preferred by coyotes (Gosselink et al., 2003; McDonald et al., 2008).

Free‐ranging domestic dogs (*Canis familiaris*; hereafter “dogs”) are commonly associated with anthropogenic habitats and can further complicate competition dynamics among coyotes and foxes (Doherty et al., 2017). As subsidized predators, dogs can occur at high densities facilitating disease transmission to native canids (Acosta‐Jamett et al., 2011; Kat et al., 1995), or interfere with native canids by inducing fear‐mediated or aggressive behavior (Zapata‐Ríos & Branch, 2016). Foxes increase vigilance in the presence of dogs, (Vanak & Gompper, 2009), which can in turn increase physiological stress (Clinchy et al., 2013), susceptibility to disease (Hing et al., 2016), and decrease fitness (Pyke et al., 1977). Alternatively, dogs may indirectly provide refuge for foxes in anthropogenic landscapes by discouraging coyote activity. Coyote–dog interactions are complex and vary from playful to antagonistic and predatory (Boydston et al., 2018). Coyote avoidance of dogs could potentially reduce competition and threats of intraguild predation to foxes closer to human‐associated habitats or intensify competition among native canids in dog‐free areas.

**FIGURE 1:**
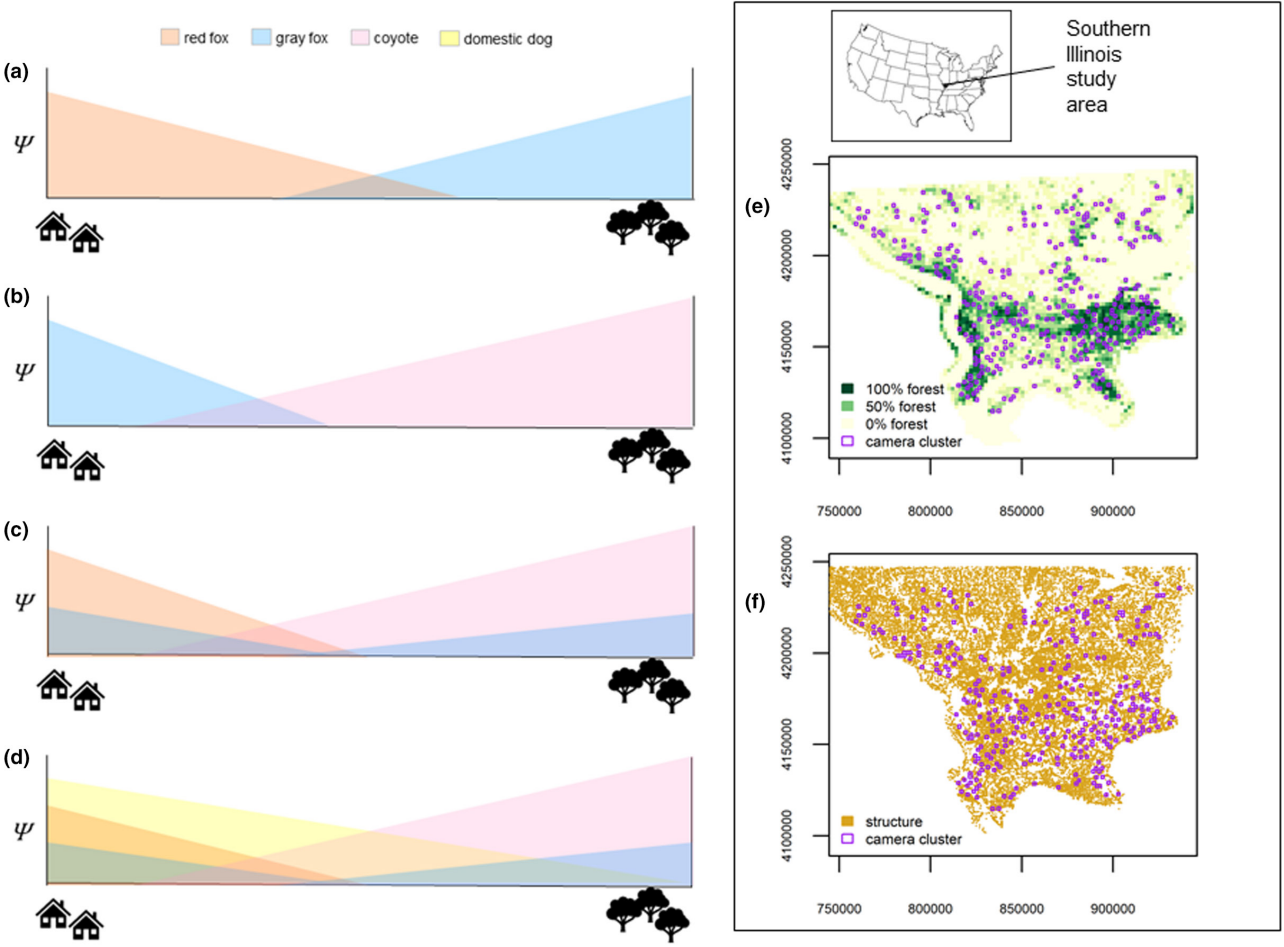
Expected patterns of occupancy among red foxes, gray foxes, coyotes, and domestic dogs (left column). When only red and gray foxes occur (a), the two similar sized species are expected to spatially segregate with red foxes exploiting more resources in more open habitats closer to human structures and gray foxes primarily foraging in deciduous forest with trees providing refugia and openings and edges providing food resources. In parts of North America where only coyotes and gray foxes occur (b), gray foxes may shift to more anthropogenic habitats to reduce interactions with coyotes. When red foxes, gray foxes, and coyotes are all present (c), gray fox occupancy declines in the forest but is limited closer to human influences by exploitative competition with red foxes resulting in an overall decline in gray fox occupancy. Domestic dogs could further limit the occupancy of both fox species (d), dependent on the distance they commonly occur from human structures. Camera trap clusters (shown in right column) were established across southern Illinois, USA, along a gradient of % forest land cover (e), and additional covariates were derived based on cluster location relative to human structures (f)

Harvest rates and bowhunter survey data from 1992 to 2015 suggest gray fox populations in Illinois declined while coyote indices increased to ubiquity (Bauder et al., 2020; Bluett, 2013; Gosselink et al., 2007; Lesmeister et al., 2015). Harvest‐based indices also suggest red fox populations have declined or remained stable in Illinois, depending on how indices were adjusted to account for confounding factors, but the raw declining trend may be an artifact of reduced trap susceptibility as red foxes increase use of urban areas (Bauder et al., 2020). In southern Illinois where overall forest cover is greater, gray fox occupancy would be expected to be greater than red fox, but instead, the estimated occupancy was nearly equivalent (gray foxes = 0.29 ± 0.03 SE and red foxes = 0.26 ± 0.04). Both gray and red fox occupancy was positively associated with anthropogenic features, suggesting high potential for exploitative competition between these species. Conversely, coyote occupancy was negatively correlated with human‐associated features, indicating both foxes may experience refuge or a shielding effect (Lesmeister et al., 2015). However, estimated occupancy throughout the study area was far greater for coyotes (0.95 ± 0.03) compared with either fox species. Local extinction probabilities ($\hat{\varepsilon }$) were greater than colonization probabilities ($\hat{\gamma }$) over 2 years for both fox species (gray fox: $\hat{\varepsilon }$ = 0.57, $\hat{\gamma }$ = 0.16; and red fox: $\hat{\varepsilon }$ = 0.35, $\hat{\gamma }$ = 0.06; Lesmeister et al., 2015) suggesting range contractions and potential population declines, with gray fox local extinction probabilities nearly twice as high as red fox. Free‐ranging dogs were common in and near human‐associated habitats with high occupancy rates across the study area (0.59 ± 0.09; Morin et al., 2018). While images of dogs in this study appeared to be healthy and homed, they rarely occurred in the company of humans (Morin et al., 2018). Thus, as gray and red foxes increase the use of areas adjacent to rural farms and other anthropogenic features to avoid coyotes, they may instead contend with dogs (Figure 1).

We used a large‐scale camera‐trap study in the southern Illinois to examine competitive interactions among native canids and free‐ranging dogs in a heterogeneous landscape. We estimated pairwise co‐occurrence and temporal overlap among canid species across the region in relation to anthropogenic features. We also used a fixed‐effect hierarchical community occupancy model to examine co‐occurrence relationships with landscape covariates conditional on multispecies occupancy. We expected both landscape context and a diverse canid community to influence competitive interactions among species (Figure 1). Based on niche theory, competing species must segregate along at least one niche axis for stable coexistence to occur (Brown, 1989; Gause, 1934; Schoener, 1974). Previous studies have demonstrated high dietary niche overlap among the three native canids (Cunningham et al., 2006; Hockman & Chapman, 1983; Masters & Maher, 2022; Neale & Sacks, 2001) including within the study area (Cypher, 1993). Thus, if niche segregation was occurring, we expected either gray and red fox occupancy or temporal overlap would be negatively associated to reduce exploitative competition, and gray foxes would exhibit spatial and temporal avoidance of coyotes to reduce intraguild interactions. Temporal overlap and spatial co‐occurrence among any of the species would indicate a lack of ecological niche segregation and would suggest at least one population is declining. Based on the documented trends in the region, we expected coyotes to be the dominant competitor to both fox species. Gray foxes are declining more rapidly than red foxes (Bauder et al., 2020) suggesting asymmetrical competitive interactions with gray foxes suffering the greatest negative interactions with coyotes via interference competition and increased exploitative competition with red foxes when avoiding coyotes. We expected dog occupancy to negatively affect native canid occupancy, especially gray foxes and coyotes, but could provide a shielding effect from coyotes.

## 2 METHODS

### 2.1 Study area

We analyzed data from a camera‐trap survey implemented across 16 counties (16,058 km^2^) in southern Illinois, USA during 2008–2010 (see Lesmeister et al., 2015 for details). The region consists of a patchwork of landcovers including agricultural croplands (44%), forest (20%), wetlands (9%), grasslands and pastures (19%), and open water (5%), with human development and urban land uses (4%) interspersed (Lesmeister et al., 2015). The area is rural and the distribution of forest and other landcover types produced a fragmented mosaic landscape, (Figure 1) with relatively high edge density (McDonald et al., 2008). Public land holdings (approximately 12% of the study area) were generally aggregated but not contiguous and are permeated by private inholdings. Human land use was pervasive with nearly 50% of the region covered by agriculture or urban land uses (Figure 1). Across the region, 80% of 30 m × 30 m pixels were within 1 km of human structures (Morin et al., 2018) and most of the nearest human structures to camera‐traps were rural houses or agriculture buildings (Figure 1; Lesmeister, 2013).

### 2.2 Camera‐trap survey

We employed a stratified random sampling approach to select 357 grid cells (2.6‐km^2^ each) within the study area with forest cover ranging from 11% to 100% (Lesmeister et al., 2015). We deployed clusters of digital remote cameras (Cuddeback Excite [2.0 megapixel] or Capture [3.0 megapixel] digital remote cameras; Non Typical, Inc., Park Falls, WI) within grid cells for one three‐week session conducted January–April. We established a cluster of 3–4 baited camera stations ≥250 m apart within each cell, resulting in 1188 stations surveyed. It was not possible to survey all camera trap clusters in a single year; thus, we stratified survey effort and operated one‐third of the camera trap clusters in 2008, another third in 2009, and the last third in 2010. As a result, each camera trap cluster was sampled for one three‐week session over the three‐year study. We treated each camera trap cluster as a sampling unit for occupancy analyses described below to improve detection and reduce the influence of baiting (Kolowski et al., 2021).

### 2.3 Statistical analyses

We used three approaches to investigate potential interactions among native canids and dogs across the study area. First, we used the $\psi^{\mathrm{BA}}$ parameterization of a single‐season two‐species co‐occurrence model (MacKenzie et al., 2004; Richmond et al., 2010) to make pairwise assessments of conditional occupancy and detection rates among canids. Using the conditional occupancy parameterization, we were able to explicitly assess support for hypotheses regarding directional interactions among canid species. Second, we used temporal kernel density estimators to quantify diel activity pattern overlap among pairs of canids (Ridout & Linkie, 2009). Third, we fit a fixed‐effects hierarchical community occupancy model (Dorazio & Royle, 2005) with the four canid species and including landscape covariates with greatest support from the two‐species co‐occurrence models; we partitioned the resulting posterior distributions to compare occupancy probabilities of gray foxes conditional on the occupancy state of combinations of the other three canids in a single multispecies analysis to assess support for hypothesized indirect and direct interactions.

#### 2.3.1 Two‐species co‐occurrence

We used the $\psi^{\mathrm{BA}}$ parameterization (Richmond et al., 2010) of the two‐species co‐occurrence model to assess support for hypotheses related to pairwise canid interactions (Appendix A). An alternative to the phi/delta co‐occurrence parameterization described in MacKenzie et al. (2004), the $\psi^{\mathrm{BA}}$ parameterization estimates occupancy and detection of a focal species (species B) conditional on occupancy and detection of an interacting species (species A) and is more numerically stable with the addition of covariates.

The model includes three estimated occupancy parameters ($\psi^{A}$: the probability of occupancy for interacting species A, $\psi^{\mathrm{BA}}$: the probability of occupancy for focal species B when species A is present, and $\psi^{\mathrm{Ba}}$: the probability of occupancy for focal species B when species A is absent). Parameters $\psi^{\mathrm{BA}}$ and $\psi^{\mathrm{Ba}}$ can be constrained to be equal or estimated separately, and comparing fit of these two options (e.g., via information theoretic model selection) can provide inference as to whether occupancy of species B is affected by the occupancy state of species A, while co‐occurring ($\psi^{\mathrm{AB}}$).

Five detection parameters also are estimated: $p^{A}$, the probability of detection for species A, given species B is absent; $p^{B}$, the probability of detection for species B, given species A is absent; $r^{A}$, the probability of detection for species A, given both species are present; $r^{\mathrm{BA}}$, the probability of detection for species B, given both are present and species A is detected; and $r^{\mathrm{Ba}}$, the probability of detecting species B, given both species are present and species A is not detected. These detection parameters can be constrained or estimated separately to form competing hypotheses assessing whether probability of detection of either species is independent of or conditional on the detection or occupancy state of the other species. In addition to correcting for potential biases in occupancy estimates resulting from unmodeled heterogeneity in detection, support for detection of a species being conditional on the state of the other species can suggest more subtle forms of interactions including a behavioral response (attraction or avoidance) or differences in local densities, as detection rates can be related to number of individuals available to be detected (Royle & Nichols, 2003).

**FIGURE 2:**
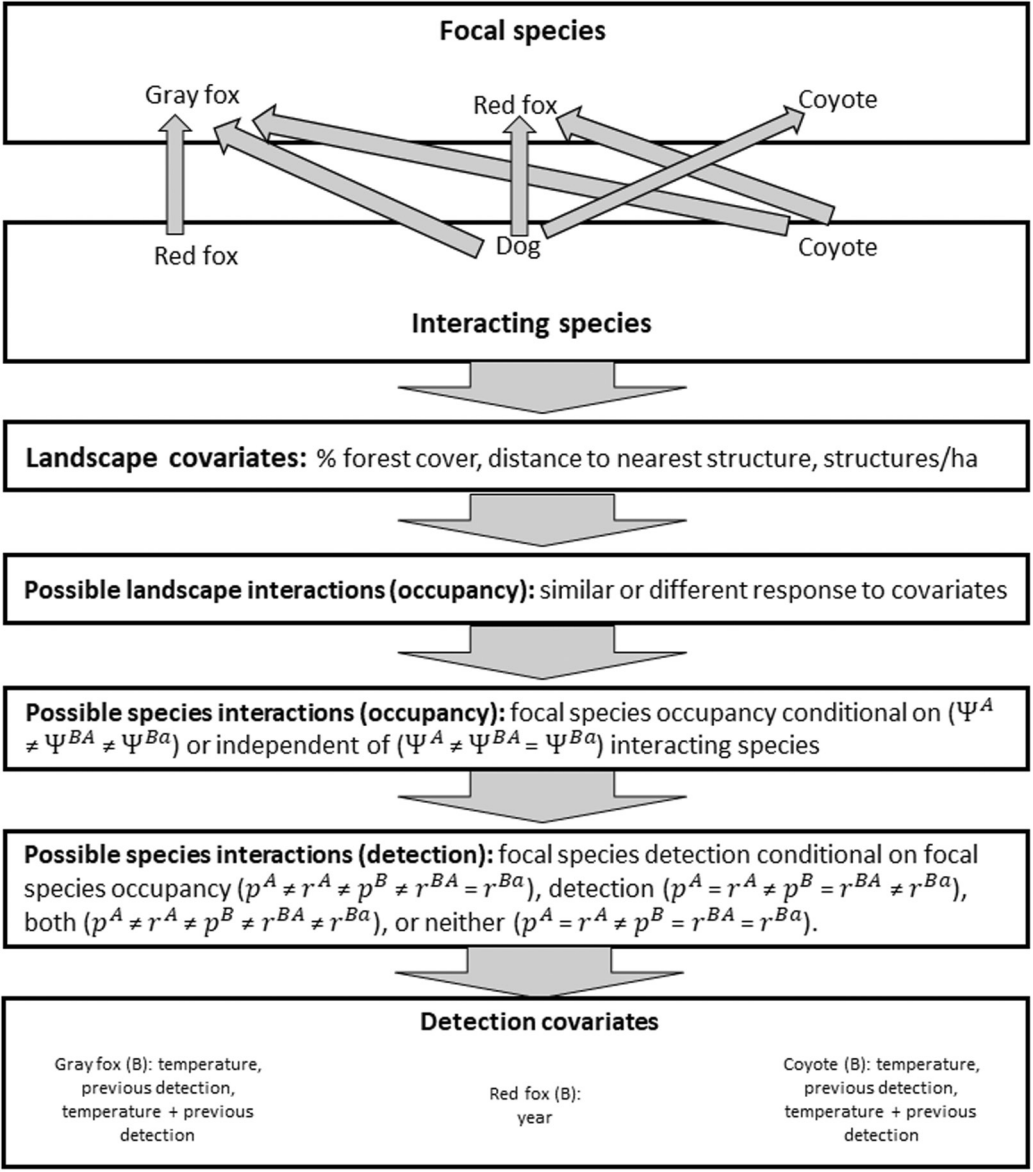
Progression through 2‐species conditional occupancy candidate set to evaluate co‐occurrence among canid species in southern Illinois, USA in relation to landscape covariates (% forest, distance to nearest structure, and structures/ha). The candidate set compared occupancy sub‐models with differential response to landscape features or response constrained to be the same, and models with focal species (species B) occupancy conditional on or independent of the interacting species (species A). The candidate set included comparisons of detection sub‐models where detection of species A and B were independent, detection of species B was conditional on species A occupancy, detection, or both. Covariates in the detection sub‐models were selected based on previous support in single‐species occupancy models for species B

We considered six pairings of focal and interacting species (Figure 2) resulting in six candidate model sets. We assigned gray foxes as the focal species (B) in the first two pairs to assess how interactions with native canids (red foxes or coyotes) may be contributing to population declines. We assigned red foxes as the focal species interacting with the coyotes to assess potential for negative impacts of the larger predator on red fox distribution. Finally, we assigned dogs as the interacting species (A) in the remaining three interactions (with gray foxes, red foxes, and coyotes) to assess how the introduced species may be impacting occupancy and responses of native canids as the focal species.

For each species pairing, we used previously supported detection covariates for focal species B to estimate its occupancy conditional on the occupancy of the interacting species (A), and the detection of species B as conditional on the occupancy state or detection of species A. We compared models where occupancy of species B was independent of ($\Psi^{A}$ ≠ $\Psi^{\mathrm{BA}}$ = $\Psi^{\mathrm{Ba}}$), or conditional on the presence of species A ($\Psi^{A}$ ≠ $\Psi^{\mathrm{BA}}$ ≠ $\Psi^{\mathrm{Ba}}$). We selected three landscape covariates with support from previous studies in Illinois (Cooper et al., 2012; Lesmeister et al., 2015; McDonald et al., 2008; Morin et al., 2018), and suspected influence on interactions among candid competitors: % forest land cover within 0.40 km of a camera station representing 20% of a gray fox home range (Lesmeister et al., 2015), distance to nearest human structure, and density of structures/ha. We compared models where species responded similarly to each landscape covariate with models with species‐specific responses to the covariate (interaction term) and models with no landscape covariates. For detection models, we included combinations of mean weekly survey temperature and previous detection of species B (gray foxes or coyotes), or year the camera cluster was operational survey (one of three possible years; red foxes) as detection covariates. We compared models where detection of species was independent of the other species ($p^{A}$ = $r^{A}$ ≠ $p^{B}$ = $r^{\mathrm{BA}}$ = $r^{\mathrm{Ba}}$) to models where detection was dependent on presence of the other species ($p^{A}$ ≠ $r^{A}$ ≠ $p^{B}$ ≠ $r^{\mathrm{BA}}$ = $r^{\mathrm{Ba}}$), models where occupancy of species B was dependent in the detection of species A ($p^{A}$ = $r^{A}$ ≠ $p^{B}$ = $r^{\mathrm{BA}}$ ≠ $r^{\mathrm{Ba}}$), and models where detection is conditional on occupancy of the other species and detection of species B is conditional on detection of species A ($p^{A}$ ≠ $r^{A}$ ≠ $p^{B}$ ≠ $r^{\mathrm{BA}}$ ≠ $r^{\mathrm{Ba}}$).

We fit two‐species co‐occurrence models in R (R Core Team, 2016) using the RPresence package (v2.12.7; MacKenzie & Hines, 2017) and compared support for models with Akaike's Information Criterion (adjusted for small sample size; AICc) and relative Akaike model weights ($w_{i}$; Burnham & Anderson, 2002). We considered Akaike weights for models within 10 ΔAICc of the top model and considered any models with within 2 ΔAICc to be competing, in that one competing model is not supported over another (Table A1). We derived probability of co‐occurrence ($\Psi^{\mathrm{AB}}$ = $\Psi^{A}\Psi^{\mathrm{BA}}$; Richmond et al., 2010) and calculated the standard errors by taking the square root of the variance of two random variables ($\Psi^{A}$ and $\Psi^{\mathrm{BA}}$; Mood et al., 1974).

#### 2.3.2 Multi‐species hierarchical community occupancy model

We used a fixed‐effects multi‐species hierarchical community occupancy model (Dorazio & Royle, 2005) to simultaneously investigate co‐occurrence among the four canid species in response to habitat covariates and accounting for imperfect detection. By combining data for all four species in a single model, we were able to partition posterior distributions and compare gray fox occupancy probabilities in the presence of combinations of the other three canids (dogs and coyotes, red foxes and coyotes, red foxes and dogs, and all three), explicitly testing for spatial niche segregation which would be suggested by lower gray fox occupancy probabilities in the presence of the other species. We included % forest and distance to structure as covariates for occupancy and mean temperature as a covariate for detection based on common support among multiple canids in single‐species occupancy and co‐occurrence results (Lesmeister et al., 2015; Morin et al., 2018). We used Markov‐Chain Monte Carlo (MCMC) sampling to estimate posterior distributions of parameters (detection, occupancy, species richness at each site, and beta coefficients) in JAGS (version 4.3.0; Plummer, 2003) using a wrapper package (jagsUI version 1.5.0; Kellner, 2018) in R. We fit the model with three MCMC chains including an adapt phase of 10,000, 5000 burn‐in, 15,000 iterations, no thinning, and checked the sensitivity of output to the normal prior distribution on occupancy (Northrup & Gerber, 2018). We assessed convergence with visual inspection of trace plots, effective sample size estimates, and Rubin‐Gelman diagnostic ($\hat{R}$) <1.1 (Gelman & Rubin, 1992). We saved the z‐matrix for each species for each iteration (a 1 or 0 indicating whether a species was predicted to occur or not occur at a site) to allow for post‐processing of posterior distributions of site occupancy to evaluate conditional occupancy probabilities among species. We estimated mean and median number of species per site and compared beta coefficients for % forest and distance to structure for each species compared to the community mean to compare covariate relationships among canids.

#### 2.3.3 Temporal overlap

We estimated temporal activity and overlap among canids (six pairwise comparisons) using temporal kernel density estimates in the R package overlap (version 0.3.3; Ridout & Linkie, 2009). We defined a single event as all photographs of a species within a 30‐minute window to allow for independence of events (Di Bitetti et al., 2006). We used a nonparametric kernel density overlap estimator $\hat{\Delta_{4}}$ to produce unbiased estimates of activity overlap as was appropriate for large sample sizes (Ridout & Linkie, 2009). This quantitative measure ranged from 0 to 1 for no overlap to complete overlap in activity patterns. We calculated confidence intervals for overlap estimates using bootstrap resamples with the “basic0” bias correction. We also divided activity data for each species into two sets based on distance to anthropogenic structures: camera stations closer than the median distance (409.48 m; “near” set), and camera stations farther than the median distance (“far” set), and estimated overlap between the two sets for the same species to coarsely assess if temporal activity was different based on proximity to human structures.

## 3 RESULTS

We collected 102,711 photographic detections of endothermic animals over 29,988 camera trap days at the 357 camera clusters surveyed for three weeks and binned into one‐week occasions. Of the canid guild, we recorded the greatest number of detections of coyotes (485) and dogs (334), and fewer of gray foxes (117) and red foxes (76).

### 3.1 Two‐species co‐occurrence

**FIGURE 3:**
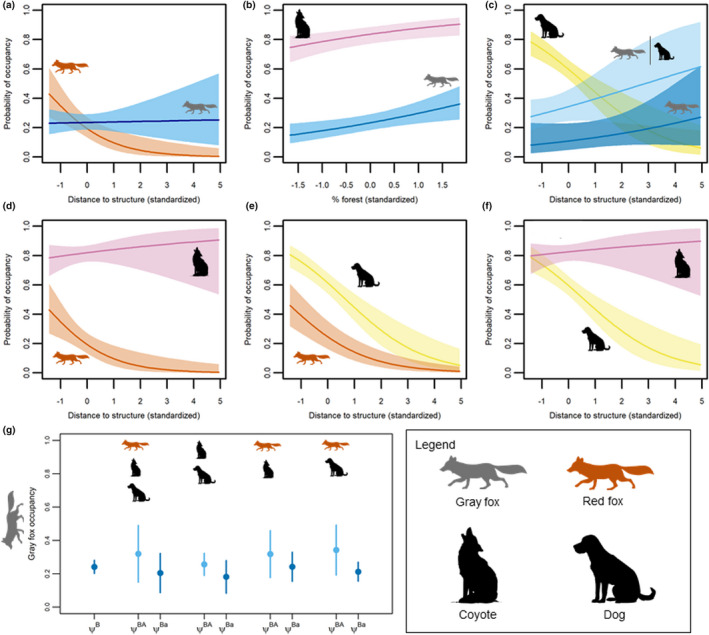
Predicted and estimated canid occupancy in southern Illinois, USA. Predicted occupancy (mean and 95% CI) based on top‐ranked two‐species conditional occupancy models for each pairwise comparison including (a) red fox – gray fox, (b) coyote – gray fox, (c) domestic dog – gray fox, (d) coyote – red fox, (e) dog – red fox, and (f) dog – coyote. Occupancy (*y*‐axis) is predicted over the landscape covariate with the greatest model selection support for the pairwise candidate set (*x*‐axis: distance to structure or % forest land cover). Multispecies occupancy model posterior distributions (g; mean and 95% credible intervals) are shown for gray fox occupancy (*y*‐axis) for all sites ($\Psi^{B}$: gray fox occupancy), and when occupancy of other canids was >0.50 ($\Psi^{\mathrm{BA}}$: gray fox occupancy when other canids present) or <0.50 ($\Psi^{\mathrm{Ba}}$: gray fox occupancy when other canids absent)

Full details of model selection, estimates, and effect sizes for two‐species occupancy models are included in the Appendix A. Overall, evidence indicated gray fox occupancy was independent of red fox occupancy after accounting for their differing responses to proximity to structures (Figure 3), and gray fox detection was higher ($\hat{p^{B}}$ = 0.47 ± 0.04) and not conditional on red fox occupancy or detection ($\hat{p^{A}}$ = 0.32 ± 0.05). Gray fox occupancy and detection probabilities were lower than for coyote, but not conditional on coyote occupancy or detection, and we found no evidence for spatial segregation between gray foxes and coyotes with a positive effect of % forest cover on occupancy for both species (Figure 3). Red fox occupancy and detection were also lower and independent of coyote presence and coyote detection ($\hat{p^{A}}$ = 0.55 ± 0.02) was higher than red fox detection ($\hat{p^{B}}$ = 0.32 ± 0.05).

Gray fox occupancy was higher when dogs were also present, but red fox and coyote occupancy were independent of dog occupancy (Table A1). Red fox occupancy was lower than dogs across the gradient of distance to structures. Conversely, coyote occupancy was consistently higher than dog occupancy and their co‐occurrence was greatest closer to structures ($\hat{\Psi^{\mathrm{AB}}}$ = 0.05 ± 0.18–0.63 ± 0.25 with decreasing distances). Detection probabilities of all three native canids differed with dog occupancy. Gray fox detection was lower when dogs were present ($\hat{p^{B}}$ = 0.60 ± 0.06, $\hat{r^{\mathrm{BA}}}$ = $\hat{r^{\mathrm{Ba}}}$ = 0.40 ± 0.05), and similarly dog detection was lower when both species were present ($\hat{p^{A}}$ = 0.58 ± 0.03, $\hat{r^{A}}$ = 0.39 ± 0.05). Red fox detection changed with sampling year and was higher when dogs were also present ($\hat{r^{\mathrm{BA}}}$ = $\hat{r^{\mathrm{Ba}}}$ = 0.28 ± 0.06 in 2008, 0.24 ± 0.05 in 2009, 0.42 ± 0.06 in 2010) compared to when they were absent ($\hat{p^{B}}$ = 0.18 ± 0.05 in 2008, 0.15 ± 0.05 in 2009, 0.30 ± 0.07 in 2010). Dog detection was also higher when red foxes were present ($\hat{r^{A}}$ = 0.60 ± 0.05 in 2008, 0.54 ± 0.05 in 2009, 0.74 ± 0.05 in 2010 vs. $\hat{p^{A}}$ = 0.46 ± 0.05 in 2008, 0.40 ± 0.04 in 2009, 0.61 ± 0.04 in 2010). Detection of dogs and coyotes decreased with increased temperature ($\hat{\beta }$ = −0.13 ± 0.06) and coyote detection was higher when dogs were present ($\hat{r^{\mathrm{BA}}}$ = 0.52 ± 0.04–0.65 ± 0.04, $\hat{r^{\mathrm{Ba}}}$ = 0.40 ± 0.05–0.54 ± 0.05), while dog detection was unchanged by coyote presence ($\hat{p^{A}}$ = $\hat{r^{A}}$ = 0.45 ± 0.04–0.60 ± 0.04).

### 3.2 Multi‐species hierarchical community occupancy model

The mean canid species richness at a site was 1.88 ± 0.77. Species richness decreased slightly with increased distance to structures ($\hat{\beta }$= −0.34, 95% CRI = −1.32 – 0.65) while there was little support for an effect of % forest ($\hat{\beta }$ = 0.08, 95% CRI = −0.66 – 0.84). Based on the posterior estimates for species richness for each site, median species richness was 2.17 within the 1st quartile of distances to structure (closest to human structures) and declined to 1.17 for the last quartile (farthest from human structures). Median species richness was similar for the 1st (2.08), 2nd (2.09), and 3rd quartiles (2.10) of % forest, but declined slightly (1.70) in the last quartile (sites with the greatest % forest cover). Point estimates of gray fox occupancy was consistently higher when other canids were also present (Figure 3) demonstrating a lack of spatial niche segregation.

### 3.3 Temporal overlap

**FIGURE 4:**
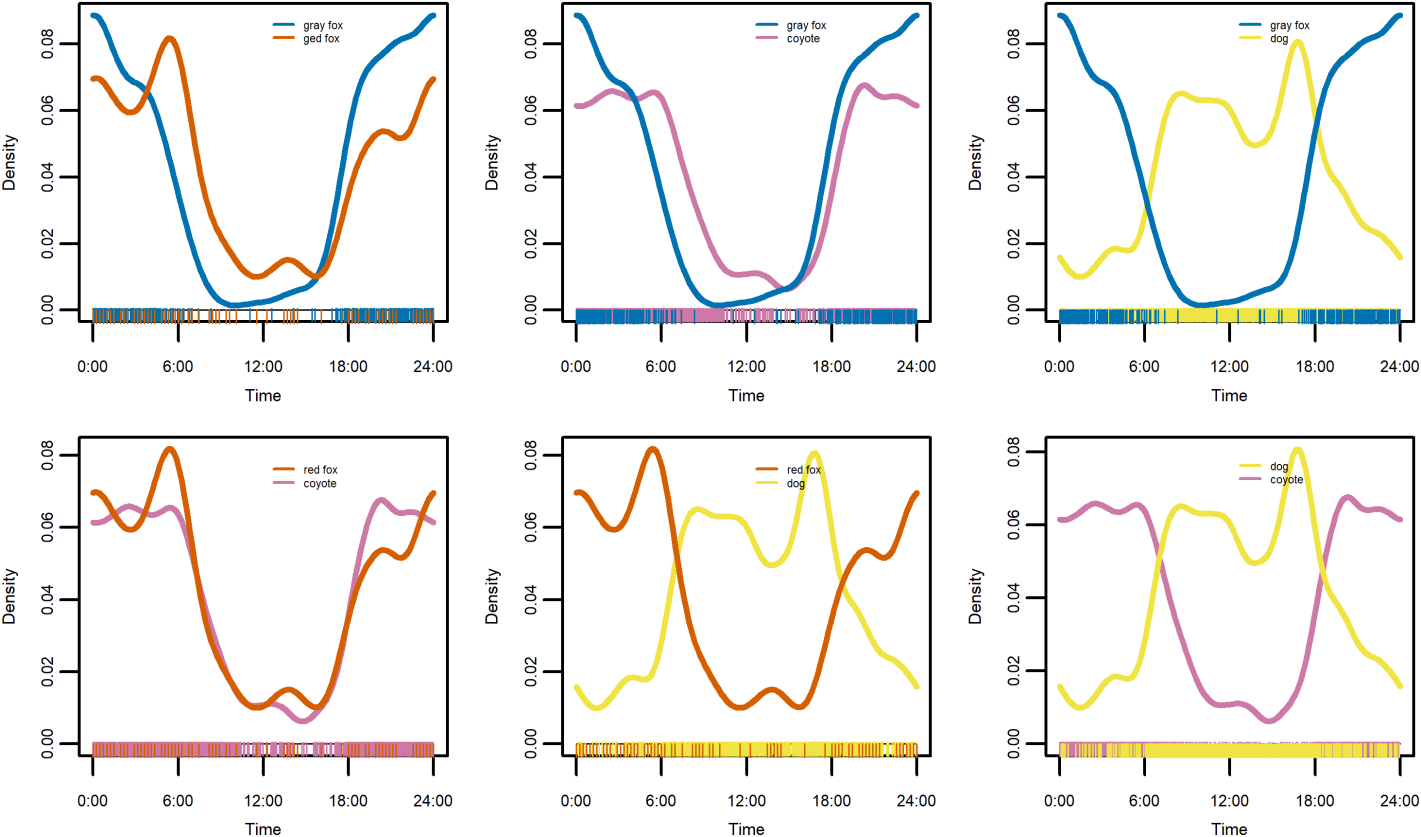
Temporal activity patterns and overlap for pairwise comparisons among canid species (clockwise from top left: red fox – gray fox, coyote – gray fox, domestic dog – gray fox, dog – coyote, dog – red fox, coyote – red fox). Activity is represented by the estimated kernel density (*y*‐axis) of number of photographic events for each species over a 24‐h period (*x*‐axis)

Temporal activity overlapped substantially among the three native canid species, all of which demonstrated nocturnal and crepuscular activity patterns (Figure 4). Temporal overlap ($\hat{\Delta_{4}}$ with 95% CI) was greatest between coyotes and red foxes (0.93, 0.87–0.98), lower between coyotes and gray foxes (0.83, 0.79–0.88), and least between red foxes and gray foxes (0.79, 0.70–0.87). Dogs exhibited a diurnal activity pattern and pairwise overlap between dogs and native canids was reduced. Overlap with dogs was less for gray foxes (0.40, 0.35–0.45) compared to coyotes (0.50, 0.46–0.53) and red foxes (0.50, 0.42–0.57). Activity patterns were similar at camera stations close to structures and far from structures for all species (gray fox $\hat{\Delta_{4}}$ [with 95% CI] = 0.95, 0.88–1.00; red fox $\hat{\Delta_{4}}$ = 0.88, 0.74–0.99; coyote $\hat{\Delta_{4}}$ = 0.95, 0.92–0.99; dog $\hat{\Delta_{4}}$ = 0.92, 0.87–0.96).

## 4 DISCUSSION

Intraguild interactions among predators are complex and nuanced, with coexistence hinging partly on landscape configuration and habitat quality (Gompper et al., 2016). At the scale of our analysis, we found differential habitat use but no evidence of avoidance among native canids in our study area suggesting opportunities for spatial and temporal segregation are limited by low‐grade but pervasive presence of humans. The perpetual disadvantage to gray foxes created by asymmetrical competitive interactions with coyotes and red foxes in this context substantiates concerns of population declines throughout the Midwest United States (Allen et al., 2021; Bauder et al., 2020; McTaggart, 2018) and highlights limitations of anthropogenic benefits to mesopredators when niche overlap is high (Jachowski et al., 2020). The unusually high occupancy probability of dogs (Morin et al., 2018) may exacerbate impacts to gray foxes by increasing competitive pressures among native canids. The culmination of these factors may foreshadow declines of currently common species over time and reductions in diversity as rising human densities and sprawl crowd out the available refuge and productivity of habitat complexity found in wildlands (Manlick & Pauli, 2020; Oliver et al., 2010; Olivier et al., 2020).

For many wildlife species, the suitability of a landscape declines with increased intensity, duration, and extent of the human footprint (McKinney, 2006; Newbold et al., 2015), threatening persistence of species even when land esthetics are preserved (McShea et al., 2007). Extent of landcover alone does not adequately measure the effects of habitat degradation including reductions in prey, cover, and increased interspecific interactions (Maerz et al., 2009; Smith et al., 2018). While gray fox occupancy was positively correlated with amount of forest landcover, occupancy was still low even when forest landcover was 100% ($\hat{\Psi }=$ 0.36 ± 0.06). In this study area, Lesmeister et al. (2015) indicated gray fox local site and core home range use increased with coarse woody debris and landcover edge density—habitat and landscape metrics associated with greater prey availability in forests (Anderson et al., 2003; Fauteux et al., 2012). Landscape complexity can also reduce the frequency of interactions with coyotes and influence prey use (Gulsby et al., 2017, Ward et al., 2018), whereas reductions in complexity coupled with widespread human presence can increase competitive interactions (Parsons et al., 2019).

Co‐occurrence is not synonymous with coexistence. The effects of competition occur dynamically along one or many niche axes, and competitive exclusion can be a slow and persistent process (Yackulic, 2017). Species can co‐occur and still demonstrate long‐term trends of population decline and local extirpation for one species (Yackulic et al., 2019). Niche segregation is required to minimize competition, but we found no evidence of spatial or temporal avoidance among the three native canids suggesting broad niche overlap setting the stage for intensified competition. For example, while gray fox occupancy was not conditional on coyote occupancy, both displayed the same occurrence trend relative to % forest cover except coyote occupancy was consistently much higher than gray fox (Figure 3). Given gray fox population declines in the region (Allen et al., 2021, Bauder et al., 2020), it is reasonable to conclude the simultaneous increase in coyote occurrence has reduced gray fox occurrence steadily over time across the landcover gradient, and not that they co‐occur in stable populations along this gradient.

Gray foxes are commonly considered a generalist species, often benefitting from urbanization and subsidized human resources (Larson et al., 2015; Rodriguez et al., 2021), so their documented decline in this context should raise alarms about functional homogenization (Olden et al., 2004) and the context‐dependent limitations of mesopredator advantage over time in human‐dominated landscapes where apex predators are extirpated (Gigliotti et al., 2020; Prugh et al., 2009; Sévêque et al., 2020). All three native canids are dietary generalists, but gray fox relative habitat specialization (predator avoidance via hiding and climbing as opposed to fleeing) appears to render the species the least generalist species of the suite and possibly the most vulnerable to changing competitive dynamics (Clavel et al., 2011).

Based on our findings, dietary generalization is less advantageous than habitat generalization when interspecific interactions are intensified. Even though red fox occupancy was low as expected based on the limited red fox habitat in the study area, red foxes have already demonstrated the capacity to replace other more specialized fox species from parts of their range, including artic foxes (*Vulpes lagopus*; Hamel et al., 2013) and sand foxes (*Vulpes ruepelli*; Ilani, 1988). The scattered but pervasive presence of humans in the study area forest may be enough for red foxes to increase competitive pressures on gray foxes. Furthermore, subordinate predators are commonly relegated to prey‐poor areas or less optimal habitat including human use areas (Steinmetz et al., 2013; Thapa et al., 2021). Gray foxes demonstrate habitat selection for human use areas outside of the red fox range when coyote activity is high (Deuel et al., 2017; Riley, 2006). Thus, gray foxes could be slowly extirpated from parts of their range when forced to co‐occur with red foxes and are unable to shift space use and activity sufficiently to reduce interference competition with coyotes (Levi & Wilmers, 2012; Palomares & Caro, 1999).

Mean richness at a site was 2 canid species, but the low richness ceiling (Wiens, 1977) may be due to trophic downgrading facilitating functional homogenization (Estes et al., 2011), and not overall resource availability. Diverse carnivore assemblages require greater discrepancy in size including large apex carnivores (Caro & Stoner, 2003; Dalerum et al., 2009) long extirpated from the study area. Coyotes were ubiquitous in our study area but shift areas of activity when larger intraguild predatory wolves (*Canis lupus*) are present (Ripple et al., 2013) which could provide increased niche space for gray fox. Red fox populations are known to benefit from the presence of wolves (Levi & Wilmers, 2012; Newsome & Ripple, 2015), and gray foxes will intentionally acquire scent from puma (*Puma concolor*) scrapes to deter coyotes (Allen et al., 2017). Large carnivore populations are recovering and expanding in North America and there are indications of possible human support for large carnivores in the region (Smith et al., 2014). However, available habitat for wolves, black bears (*Ursus americanus*), and pumas in southern Illinois is small and isolated (Smith et al., 2016).

Our findings suggest dogs may compound demographic impacts to gray foxes in our study area. While gray foxes occurred at higher rates when dogs were also present, diel activity patterns differed substantially, and gray fox detectability was lower when dogs were present indicating either avoidance (including suppression of activity) or lower gray fox abundance in the areas where they co‐occur with dogs (Royle & Nichols, 2003). Whether through disease transmission or increased vigilance reducing individual fitness, either interaction could contribute to a population decline (Doherty et al., 2017; Sheriff et al., 2009). However, the greater consequence could be that the high occurrence of dogs in the study area escalates competitive interactions among native canids. Dogs could heighten exploitative competition among red and gray foxes by killing or scaring prey and the increased gray fox occupancy when dogs were present and similar response of dog and red fox occupancy to anthropogenic features suggest increased overlap in spatiotemporal foraging activity (Vanak & Gompper, 2009). We found no evidence that coyotes spatially avoided dogs, but the two species had considerable difference in diel activity patterns that may have ameliorated some potential negative interactions. Coyotes were detected at higher rates when dogs were detected, suggesting shared use of trails and potential for dogs to attract coyote activity in human areas where gray foxes demonstrated increased co‐occurrence with dogs. Although the high occupancy of dogs in our study area is unusual in the United States, the possible intensified competition among native canids may foreshadow outcomes of continued competitive dynamics as humans further encroach and degrade habitat and exclusion plays out (Clavel et al., 2011).

Competitive exclusion can only be confirmed over time and more research including long‐term monitoring is needed to determine the true impacts to gray foxes in the Midwest (Allen et al., 2021). While previous research in the study area revealed high rates of local extirpation, the pattern may represent an “ecological crunch” (Wiens, 1977) if the population experienced a disease outbreak or stochastic but temporary downturn in prey resources in the 2 years between surveys. However, occupancy is a coarse monitoring metric compared to abundance (MacKenzie & Nichols, 2004), so the exceptionally high rate of local extirpation in such a short period of time is troubling and warrants further investigation. Conservationists should explicitly acknowledge the ongoing process of competitive exclusion resulting from habitat degradation and trophic downgrading instead of using static measures of co‐occurrence to infer coexistence in human‐dominated ecosystems. Revisiting and repeating co‐occurrence studies over broader temporal scales (≥10 years for this canid guild based on trends identified in Bauder et al., 2021) coupled with experimental treatments to assess species interactions (Smith et al., 2020) will provide greater insight into the effects of human development on species assemblages and allow for planning of more effective conservation strategies. Specifically, a follow‐up survey at a subset of camera trap clusters, now that more time has passed, could resolve the question of whether populations have continued to decline, rebounded, or stabilized at a new equilibrium.

## AUTHOR CONTRIBUTIONS

**Dana J. Morin:** Conceptualization (equal); data curation (equal); formal analysis (lead); methodology (equal); visualization (lead); writing – original draft (lead); writing – review and editing (equal). **Damon B. Lesmeister:** Conceptualization (equal); data curation (equal); methodology (equal); visualization (supporting); writing – review and editing (equal). **Clayton K. Nielsen:** Conceptualization (equal); methodology (equal); writing – review and editing (equal). **Eric M. Schauber:** Conceptualization (equal); methodology (equal); writing – review and editing (equal).

## CONFLICT OF INTEREST

The authors have no competing interests to declare.

## Data Availability

Detection histories, covariates, and R code are available through Dryad at https://doi.org/10.5061/dryad.x95x69pmr.

## APPENDIX A Two‐species co‐occurrence model selection and results

### RED FOX–GRAY FOX

Models including effects of distance to anthropogenic structures, and an interaction term describing a differential response of the two species to distance to structure received greatest support ($w_{i}$ = 0.65; Table A1). Evidence indicated that gray fox occupancy was independent of red fox occupancy after accounting for their differing responses to proximity to structures (inclusion of a conditional effect was consistently an uninformative parameter for the same model without the interaction). There was limited support for a differential response to % forest among species ($\sum w_{i}$ = 0.20) and far less support for models including structures/hectare ($w_{i}$= 0.01), suggesting proximity to humans was more influential than density of humans in an area. Red fox occupancy probability was highest and greater than gray fox occupancy at close distances to structures ($\psi^{A}$ = 0.43 ± 0.09) but declined with greater distances to structures ($\psi^{A}$ < 0.01 ± 0.01 at maximum distances). Gray fox occupancy did not change or increased negligibly with distance to structures (Figure 2) and exhibited greater variance at greater distances ($\psi^{B}$ = 0.23 ± 0.04 at minimum distances – 0.27 ± 0.13 at maximum distances). Overall probability of co‐occurrence was very low ($\psi^{\mathrm{AB}}$ estimates ranged from 0.10 ± 0.11 at minimum distances – < 0.01 ± 0.02 at maximum distances). There was little evidence that gray fox detection was conditional on red fox occupancy or detection, and top‐ranked detection models included only the intercept and a difference between species ($\sum w_{i}$ = 0.85). Gray fox detection ($p^{B}$ = 0.47 ± 0.04) was higher than red fox detection ($p^{A}$ = 0.32 ± 0.05).

### COYOTE–GRAY FOX

A single model received strong support ($w_{i}$ = 0.92) including a positive effect of % forest cover on occupancy for both species (β = 0.34 ± 0.12) and a negative correlation between gray fox detection and mean temperature (β = −0.25 ± 0.07). Gray fox occupancy and detection were both lower than coyote, but there was no evidence either was conditional on coyote occupancy or detection. There was no evidence for spatial segregation between gray fox and coyotes. The probability of co‐occurrence increased with % forest but was generally low ($\psi^{\mathrm{AB}}$ estimates ranged from 0.11 ± 0.14 – 0.33 ± 0.23), due to the low site occupancy of gray foxes ($\psi^{B}$ = 0.36 ± 0.06 at 100% forest cover) compared with coyotes ($\psi^{A}$ = 0.91 ± 0.03 at 100% forest cover).

### DOMESTIC DOG–GRAY FOX

Our data lent overwhelming support for dog‐gray fox models that included an effect of dog occupancy on gray fox occupancy ($\sum w_{i}$ = 0.99) and detection ($\sum w_{i}$ = 0.99). Support was divided among occupancy covariates, but models that included species‐specific responses to distance to structures received greater support ($\sum w_{i}$ = 0.63) than models that included species‐specific responses to % forest ($\sum w_{i}$ = 0.36). Dog occupancy was high closest to structures ($\psi^{A}$ = 0.79 ± 0.04), but unlike red fox, did not entirely decrease to <0.01 at greatest distances from structures ($\psi^{A}$ = 0.06 ± 0.04). Gray fox occupancy was higher when dogs were also present ($\psi^{\mathrm{BA}}$ estimates increased from 0.28 ± 0.05 to 0.62 ± 0.24 with increased distance to structures while $\psi^{\mathrm{Ba}}$ increased from 0.08 ± 0.05 to 0.27 ± 0.15). Probability of co‐occurrence was highest close to structures due to the high probability of dog occupancy close to structures ($\psi^{\mathrm{AB}}$ decreased from 0.22 ± 0.18 to 0.04 ± 0.13). Conversely, gray fox detection was lower when dogs were present ($p_{B}$ = 0.60 ± 0.06, $r_{\mathrm{BA}}$ = $r_{\mathrm{Ba}}$ = 0.40 ± 0.05), while dog detection was relatively high when gray foxes were not present, but lower when both species were present ($p_{A}$ = 0.58 ± 0.03, $r_{A}$ = 0.39 ± 0.05).

### COYOTE–RED FOX

A single model received strong support ($w_{i}$ = 0.90) describing red fox occupancy decreasing with distance to structures ($\psi^{B}$ = 0.43 ± 0.09 at close distances and <0.01 ± 0.006 and maximum distances) and independent of coyote presence which increased slightly with distance to structures ($\psi^{A}$ estimates ranged from 0.79 ± 0.05 to 0.91 ± 0.09). Probability of co‐occurrence was highest close to structures ($\psi^{\mathrm{AB}}$ estimates ranged from <0.01 ± 0.07 at minimum distances to 0.33 ± 0.26). The independent species‐specific detection sub‐model received most support ($\sum w_{i}$ = 0.92) and coyote detection ($p_{A}$ = 0.55 ± 0.02) was higher than red fox detection ($p_{B}$ = 0.32 ± 0.05).

### DOMESTIC DOG–RED FOX

No single model in the dog–red fox candidate set received majority support. The $w_{i}$ for top three models = 0.39, 0.36, and 0.39, respectively, and were all within 1.43 ΔAICc. All three models included a decrease in occupancy with increased distance to structure for both species. Based on the top‐ranked model, dog occupancy ($\psi^{A}$ = 0.81 ± 0.04 at minimum distances) was higher than red fox occupancy ($\psi^{B}$ = 0.46 ± 0.08 at minimum distances) and probability of co‐occurrence also decreased with increased distance to structures ($\psi^{\mathrm{AB}}$ 0.37 ± 0.24 at minimum distances – <0.01 ± 0.01 at maximum distances). Despite the similar response to the proximity of structures, model selection lent no support for red fox occupancy being conditional on dog occupancy. However, there was strong support for detection sub‐models including effects of sampling year and heterospecific occupancy. Red fox detection was higher when dogs were also present ($r_{\mathrm{BA}}=r_{\mathrm{Ba}}$ = 0.28 ± 0.06 in 2008, 0.24 ± 0.05 in 2009, 0.42 ± 0.06 in 2010) compared to when they were absent ($p_{B}$ = 0.18 ± 0.05 in 2008, 0.15 ± 0.05 in 2009, 0.30 ± 0.07 in 2010), and dog detection was also higher when red foxes were present ($r_{A}=0.60\pm 0.05,0.54\pm 0.05,0.74\pm 0.05\;\text{in}\;2010\;\mathrm{vs}.p_{A}$ = 0.46 ± 0.05 in 2008, 0.40 ± 0.04, 0.61 ± 0.04 in 2010)). A competing model (ΔAICc = 0.15) included the same covariates with the addition of interaction terms for the occupancy and detection sub‐models, but describing similar effects.

### DOMESTIC DOG–COYOTE

The top‐ranked model in the dog–coyote candidate set included species‐specific responses to distance to structures and a fully parameterized detection sub‐model that included: detection conditional on heterospecific occupancy and detection, effects of mean temperature during the survey, and effect of previous detection of coyotes. However, models that included previous detection of coyotes with detection also conditional on occupancy and detection of dogs did not converge, perhaps a result of overfitting a behavioral response, so $p_{A}$ was not estimated. Therefore, we removed models including previous detection of coyotes in the detection sub‐model to allow evaluation of models with conditional detection between the two species and recalculated ΔAICc and relative model weights. All models receiving support in the final model set described independent species‐specific responses to distance to structures. Coyote occupancy was higher than dog occupancy across the gradient ($\psi^{B}$ increased from 0.80 ± 0.05 to 0.90 ± 0.10 at maximum distances, while $\psi^{A}$ decreased from 0.79 ± 0.04 to 0.06 ± 0.04 at maximum distances). Co‐occurrence declined with distance to structure ($\psi^{\mathrm{AB}}$ = 0.05 ± 0.18–0.63 ± 0.25). There was strong support for models in which coyote detection was conditional on dog detection ($\sum w_{i}$ = 0.90). Based on the top‐ranked model ($w_{i}$ = 0.58), detection of both species decreased with increased temperature (*β* = −0.13 ± 0.06) and coyote detection was higher when dogs were present ($r^{\mathrm{BA}}$ = 0.52 ± 0.04–0.65 ± 0.04, $r^{\mathrm{Ba}}$ = 0.40 ± 0.05–0.54 ± 0.05), while dog detection was unchanged by coyote presence ($p^{A}$ = $r^{A}$ = 0.45 ± 0.04–0.60 ± 0.04).

**TABLE A1:** Top‐ranked models two‐species co‐occurrence models for canid candidate sets estimating occupancy (Ψ) of focal species B ($\Psi^{B}$) when the occupancy state of interacting species A ($\Psi^{A}$) is present ($\Psi^{\mathrm{BA}}$) or absent ($\Psi^{\mathrm{Ba}}$), and detection (p) independent or conditional (r) on occupancy or detection. Results for models within 10 ΔAICc of the top model are presented models including uninformative parameters are not shown). Co‐occurrence was considered independent when probability of occupancy for focal species B was constrained to be the same regardless of the occupancy state of interacting species B ($\Psi^{A}\neq \Psi^{\mathrm{BA}}=\Psi^{\mathrm{Ba}}$), and conditional if occupancy of species B was dependent of occupancy state of species A ($\Psi^{A}\neq \Psi^{\mathrm{BA}}\neq \Psi^{\mathrm{Ba}}$). Detection of species B was modeled as independent of occupancy or detection of species A ($p^{A}\neq p^{B}=r^{\mathrm{BA}}=r^{\mathrm{Ba}}$), conditional on the occupancy of species A ($p^{A}\neq p^{B}\neq r^{\mathrm{BA}}=r^{\mathrm{Ba}}$), conditional on the detection of species A ($p^{A}\neq r^{A}\neq p^{B}=r^{\mathrm{BA}}\neq r^{\mathrm{Ba}}$), or conditional on the status and detection of species A ($p^{A}\neq r^{A}\neq p^{B}\neq r^{\mathrm{BA}}\neq r^{\mathrm{Ba}}$)

Species A – Species B	Co‐occurrence submodel	Occupancy covariates	Conditional detection submodel	Detection covariates	ΔAICc	wgt^a^	*K* ^b^	neg2ll^c^
Red fox – gray fox	Independent	Distance to structure + differential species response	Independent	None	0	0.65	6	1120.25
	Independent	% forest + differential species response	Independent	None	2.67	0.17	6	1122.92
	Independent	Distance to structure + differential species response	Conditional on occupancy and detection	None	3.38	0.12	9	1117.36
	Independent	% forest + species interaction term	Conditional on occupancy and detection	None	6.22	0.03	9	1120.19
	Independent	Distance to structure	Independent	None	7.31	0.02	5	1129.63
	Independent	Structures/ha	Independent	None	9.14	0.01	5	1131.46
	Independent	% forest	Independent	None	9.26	0.01	5	1131.58
	Independent	None	Independent	None	9.59	0.01	4	1133.97
Coyote – gray fox	Independent	% forest cover	Independent	Mean temperature	0	0.92	6	2046.75
	Independent	None	Independent	Mean temperature	6.37	0.04	5	2055.18
	Independent	Distance to structure	Independent	Mean temperature	8.25	0.01	6	2055
	Independent	Structures/ha	Independent	Mean temperature	8.37	0.01	6	2055.11
	Independent	% forest + differential species response	Independent	None	9.53	0.01	6	2056.28
	Independent	% forest	Independent	None	9.6	0.01	5	2058.41
Dog – gray fox	Conditional	Distance to structure + differential species response	Conditional on occupancy	None	0	0.44	8	1819.23
	Conditional	Distance to structure + differential species response	Conditional on occupancy and detection	Previous gray fox detection	2.71	0.11	11	1815.59
	Conditional	% forest + differential species response	Conditional on occupancy	Previous gray fox detection	2.73	0.11	9	1819.85
	Conditional	% forest + differential species response	Conditional on occupancy and detection	Previous gray fox detection	2.78	0.1	11	1815.66
	Conditional	Distance to structure + differential species response	Conditional on occupancy and detection	None	2.8	0.1	10	1817.81
	Conditional	% forest + differential species response	Conditional on occupancy and detection	None	3.15	0.09	10	1818.16
	Conditional	% forest + differential species response	Conditional on occupancy	None	3.86	0.06	8	1823.09
	Independent	Distance to structure + differential species response	Conditional on dog occupancy	None	7.19	0.01	7	1828.51
	Conditional	Distance to structure + differential species response	Independent	None	8.52	0.01	7	1829.85
	Independent	Distance to structure + differential species response	Independent	None	9.24	0	6	1832.64
Coyote – red fox	Independent	Distance to structure + differential species response	Independent	None	0	0.9	6	1922.02
	Independent	Distance to structure + differential species response	Conditional on occupancy and detection	None	4.65	0.09	9	1920.39
	Independent	Distance to structure	Independent	None	8.89	0.01	5	1932.98
	Independent	Structures/ha	Independent	None	9.96	0.01	5	1934.04
Domestic dog – red fox	Independent	Distance to structure	Conditional on occupancy	Year	0	0.39	8	1666.35
	Independent	Distance to structure + differential species response	Conditional on occupancy and detection	Year	0.15	0.36	11	1660.15
	Independent	Distance to structure	Conditional on occupancy and detection	Year	1.43	0.19	10	1663.55
	Independent	Distance to structure	independent	Year	3.75	0.06	7	1672.19
Domestic dog – coyote	Independent	Distance to structure + differential species response	Conditional on detection	Mean temperature	0	0.58	8	2623.81
	Independent	Distance to structure + differential species response	Conditional on detection	None	2.11	0.2	7	2628.01
	Independent	Distance to structure + differential species response	Conditional on occupancy and detection	Mean temperature	3.78	0.09	10	2623.37
	Independent	Distance to structure + differential species response	Independent	Mean temperature	4.72	0.05	7	2630.62
	Independent	Distance to structure + differential species response	Conditional on occupancy and detection	None	5.74	0.03	9	2627.44
	Independent	Distance to structure + differential species response	Independent	None	6.43	0.02	6	2634.41
	Independent	Distance to structure + differential species response	Conditional on occupancy	None	7.63	0.01	7	2633.53
	Conditional	Distance to structure + differential species response	Conditional on detection	Mean temperature	9.84	0	8	2633.65

^a^ Akaike model weight, recalculated following removal of models with uninformative parameters.

^b^ Number of parameters.

^c^ 2 × negative log‐likelihood.
